# UGDR: a generic pipeline to detect recombined regions in polyploid and complex hybrid yeast genomes

**DOI:** 10.1186/s12859-022-05113-y

**Published:** 2022-12-21

**Authors:** Amina Bedrat

**Affiliations:** 1 Institut Curie, UPMC University, Paris, France; 2 Meiogenix, 27 Rue du Chemin Vert, Paris, France; 3 Medical College of Wisconsin, 8701 W Watertown Plank Rd, Milwaukee, USA

**Keywords:** Yeast, Polyploid, Recombination, Alleles frequency, Break induced replication (BIR)

## Abstract

**Motivation:**

In eukaryotes, homologous recombination between the parental genomes frequently occurs during the evolutionary conserved process of meiosis, generating the genetic diversity transmitted by the gametes. The genome-wide determination of the frequency and location of the recombination events can now be efficiently performed by genotyping the offspring’s polymorphic markers. However, genotyping recombination in complex hybrid genomes with existing methods remains challenging because of their strain and ploidy specificity and the degree of diversity and complexity of the parental genomes, especially in $>2n$ polyploids.

**Results:**

We present UGDR, a pipeline to genotype the polymorphisms of complex hybrid yeast genomes. It is based on optimal mapping strategies of NGS reads, comparative analyses of the allelic ratio variation and read depth coverage. We tested the UGDR pipeline with sequencing reads from recombined hybrid diploid yeast strains and various clinical strains exhibiting different degrees of ploidy. UGDR allows to plot the markers distribution and recombination profile per chromosome.

**Conclusion:**

UGDR detects and plots recombination events in haploids and polyploid yeasts, which facilitates the discovery and understanding of the yeast genetic recombination map and identify new out-performing recombinants.

**Supplementary Information:**

The online version contains supplementary material available at 10.1186/s12859-022-05113-y.

## Introduction

Recombination between the maternal and paternal homologous chromosomes is a major source of genetic diversity leading to the loss of heterozygosity (LOH) events in somatic cells and meiotic crossing-overs in germline cells [1, 2]. In both cases, the evolutionarily conserved homologous recombination pathways generate novel allelic combinations that, upon Darwinian selection, are beneficial, neutral or detrimental [3]. Defects in homologous recombination in somatic cells are a source of chromosomal rearrangements [4, 5] and of sterility in sexually reproducing organisms, since meiotic recombination between the homologous chromosomes is essential to ensure their correct segregation at the reductional meiotic division (Meiosis I) [6, 7].

The variable density of single nucleotide polymorphisms (SNP) and small insertion/deletion (Indels) along the chromosomes determines the resolution of the recombined sequence extremities. Practically, it can be achieved by the genotyping and/or sequencing of these markers and their phasing in the parental and potentially recombined cells. Previous approaches for detecting recombination events along yeasts chromosomes are based on the analysis of the re-association of parental polymorphic markers and the ploidy of the cell. For example, ssGenotyping [8], a semi-supervised clustering method, used the high-density microarray sequencing data of a parental strain and its meiosis products to determine the genome wide profile of meiotic recombination in yeast tetrads. Another method, ReCombine [9] was also developed to genotype yeast meiotic progeny and to arrange recombination events into classes using either microarray or high throughput sequencing data. ReCombine aligns the reads to a merged reference genome (S288C/YJM789) and compares the number of reference versus variant markers using the base alignment quality score. Another generalized framework, RecombineX, has been developed to genotype gametes and draw the recombination profile of tetrads in different organisms including yeasts [10]. More approaches to precisely track the recombination landscape of diploid yeasts were presented. Indeed, SNP genotyping by sequencing also allowed genotyping and mapping of the recombination events in hybrid diploid cells induced to enter into meiosis prior to returning to mitotic growth (RTG) [11]. Lastly, benefiting from long-read sequencing, MuLoYDH framework [12] was designed to study the genome dynamics and mutational landscapes of yeast diploid hybrids.

All these methods have been successfully used to analyze the recombination events in the experimental setup for which they were designed (specific S288C parent, haploids, and diploids). However, due to their specificity and hard coding, these methods are less fitted to detect and plot the recombination profile of aneuploid/polyploid and/or complex hybrid yeast genomes with different genetic background. Therefore, the need for a user-friendly unbiased method that efficiently investigate recombination in natural hybrids and polyploids with uncharacterized parents remains.

In this study, we propose an Unbiased General Detection of Recombination (UGDR) method that analyzes variations in SNP frequency to detect recombination and foster visualization of recombination tracks in natural and/or constructed hybrid yeast genomes. The detection of recombination in haploid (tetrads), diploid, aneuploidy and polyploid yeasts (limited to 4n) is achieved regardless of the ploidy of the yeast and the parental genome. UGDR also addresses the challenge of the continuous variation of the allele frequencies along with chromosomal loss and gain. We validated the performance of UGDR on published yeast genome data, corresponding to a pair of mother and daughter *Saccharomyces cerevisiae* cells isolated from meiotic return to growth (RTG) and a corresponding tetrad set of four haploid spores isolated from uninterrupted meiosis [11]. Furthermore, we assessed the performance of UGDR by comparing its output against that of similar analysis and demonstrated that UGDR achieves the state-of-arts performance for both haploids and diploids and unprecedentedly extended its use to detect potential recombinations in triploid and tetraploid hybrid clinical yeast strains.

## Methods

For the detection and the visualization of recombination, we employed a three-step method. First, we aligned the raw sequence reads to the reference genome (S288C and/or SK1). Second, we performed variants calling to identify the polymorphic sites and to characterize the normalized allelic frequency of the individual markers in the parental and recombinant samples. Third, we plotted the allelic variation to provide a quick and intuitive way to map the recombination events.

### Dataset

To test UGDR, we downloaded publicly available Illumina paired-end reads sequencing data of recombined and potentially recombined strains from the NCBI Sequence Read Archive (SRA) (Additional file 1: Table S1). The downloaded set includes: four-spore tetrads of haploid strains and two diploid mother and daughter RTG strains (RTG17M & RTG17D,) issued from a SK1/S288C hybrid background [11], and two clinical triploids strains (YJM1138 and YJM114) and two clinical tetraploids strains (YJM958, TJM959) [13]. It is important to note that the triploids and tetraploids are sequenced by Zhu et al. without sporulation or induced recombination. The S288C fasta sequence was downloaded from the Saccharomyces Genome Database and the novel SK1 fasta assemble was provided by Liti [5, 14, 15].

### Genome alignment and variants calling (Galocal)

**Fig. 1:**
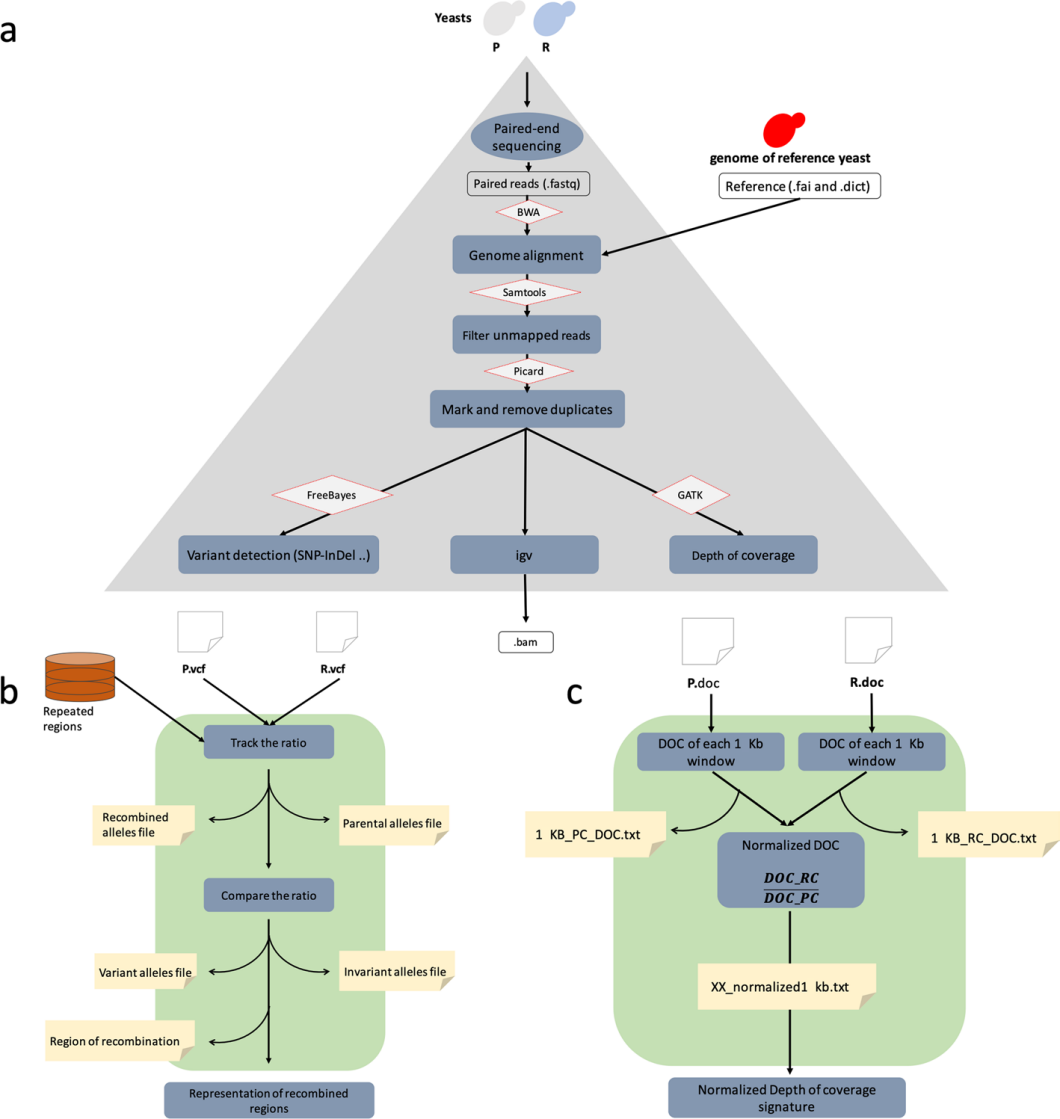
Workflow of data recombination and coverage analysis. Blue rectangles represent the methods used; yellow squares represent the output files. Lozenges represent name labels for the tools used. **a** Galocal, a pipeline for analyzing sequencing data and calculating depth of coverage. This pipeline encompasses a list of tools to analyze paired-end Illumina sequence. It allows (1) reads’ mapping, (2) filter and remove duplicates, (3) call variant and (4) calculate per base depth of coverage. At the end of this step, three text files are generated (.vcf, .doc and .bam). **b** UGDR, a pipeline to analyze recombination. This pipeline considers two VCF files as an input (one file for the Parental or reference strain P.vcf and a file for the potentially recombined strain R.vcf). (1) The pipeline calculates the ratio from the VCF files and classify them according to their origins: Parental/reference alleles file and Recombined alleles file (2) Compare the allele ratio between parent/reference and the recombined strains, univariate ratios are written in the invariant alleles file and the varying ratios are written in variant alleles file. (3) The last step is to compare the alleles ratios and stock the result in Region of recombination file. Once the files are created, recombination profile is created. **c** NDoC, a pipeline to calculate Depth of Coverage. A depth of coverage is calculated within a 1 kb window from the parental and the recombined DOC files and the results are written in two distinct files. Next, the normalized depth of coverage is calculated, and the data are stoked in xx_normalized1KB file (xx: name of the potentially recombined strains). Lastly the coverage plot per chromosome is plotted

The key steps for studying large-scale dataset are reads mapping and identification of potential polymorphic sites (SNP calling). To manage the alignment of the reads and call the variants, we assembled the necessary open source tools in a custom bash script named Galocal (Fig. 1a). First, we mapped all the reads to the reference using the gapped Burrows-wheeler Aligner (BWA) v.0.7.15 [14]. Next, to achieve an accurate variant detection, we conducted a different post-alignment analysis that included elimination of unmapped reads using SAMtools v.1.3 [14] and removal of PCR duplicate using Picard tools v.2.4.1 (https://github.com/broadinstitute/picard) [16]. After that, we used the Bayesian algorithms: FreeBayes framework-1.0.2 [17] to call variants and the DepthOfCoverage tool from the GATK v3.5 framework [18] to determine the depth of coverage (DoC). Finally, the output files (variants files [.vcf] and depth of coverage files [.doc]) were further used to analyze the variations in allelic frequency reflecting the region of recombination.

### Analysis of allele frequency variation and identification of recombination region (UGDR)

When describing the allele frequency variation in this study, we use the term “ratio variation”. To analyze ratio variation, we investigated and compared each chromosome’s copy number that supported the alleles in both the potentially recombined strains and reference strain. To start the exploration of the chromosome’s copy involved in the recombination, it must be noted that the allele (SNP) ratios are ploidy dependent (Additional file 1: Table S2). Indeed, in haploid strains, the heterozygous allele ratio is equal to 1. Meanwhile, in diploid strains, the heterozygous allele ratio is equal to 0.5 and the homozygous allele ratio is equal to 1. In triploid and tetraploid strains, the heterozygous allele ratios are 0.33, 0.67 and 0.25, 0.5, 0.75, respectively [13]. To calculate the allele ratios from the variants calling files (VCF), we involved two main constants: (1) the observed number of alternative alleles (AO), (2) and the total number of bases sequenced and aligned at a given reference base position (DP). Therefore, the ratio represents the fraction of an alternative allele’s number in the total number of bases at a given position ($Ratio= AO/ DP$). Further, the allele ratio is tightly dependent on the quality and counts of the reads at each position; so, the selected alleles for the study are those supported by enough coverage (minimum 20) and an average Phred quality score $>200$ [19] and AO to $>20$ for an 80$\times$ sequencing coverage (cutoffs that the users can adopt to their data). The ratio was calculated, for each allele passing the filters, and summarized with other information such as position and type of the polymorphism in two distinct files called “parental alleles file”, for the data issued from the reference stain, and “recombined alleles file” for the potentially recombined strain (Fig. 1b).

Next, to detect recombination, we considered the allelic ratio as: (1) invariable if the allele ratio was equal in the parental (P) and potentially recombinant (R) strains (Additional file 1: Fig. S1B), and (2) variable if the P alleles ratio was significantly different and followed the predicted likelihood of ratio variation summarized in (Additional file 1: Fig. S1-A and Table S3). For example, during recombination of a triploid cell, the variants with a parental ratio of 0.33 may vary to 0.66 or 0 (Additional file 1: Fig. S1c-top). If the ratio changes from 0.33 to 0 or 1, with a loss of depth of coverage this indicates a loss of a chromosome (3n to 2n—Additional file 1: Fig. S1D-top). In contrast, If the ratio changes from 0.33 to 0.5 or 0.25 with a gain of depth of coverage this indicates a gain of a chromosome (3n to 4n—Additional file 1: Fig. S1D-top). The outcome of this step is stored in two files called “variants alleles file” for information on the alleles with variants and “invariant alleles file” for those with invariant ratios. To robustly call the recombined region, we only retained those delimited by two break points holding at least 4 adjacent alleles that have undergone a variation of their allelic ratio. Nonetheless, regions that contain additional alleles with an invariable ratio were considered when a pair of two variant alleles were separated by a maximum of two invariant ones.

Lastly, the UGDR pipeline creates the “Region of recombination” file that contains all the information about the recombined regions (start and end as well as the number of variable alleles composing the region) and plots the recombination map.

### Calculate the normalized depth of coverage (NDoC)

To evaluate the recombination region detection analysis, several features like Depth of Coverage (DoC) and mapping quality are important. The analysis of DoC is essential to detect events like insertion, deletion and copy number variation (CNV) between and along the chromosomes during recombination. We used the program DepthOfCoverage of the GATK toolkit [18] to obtain the base-level coverage of the genome (Fig. 1c). After that, to correct the technical DoC variation between parent and recombined cells, we calculated a normalized depth of coverage by dividing the base coverage of the recombinant cell by the base coverage of the parent and averaged the DoC by a one Kb sliding window along the genome (A cutoff that the users can adapt to their data). At the end of this step, the normalized coverage is written to a “Samplename_depthofCove.txt” file and the Depth of coverage plot is generated.

### Display of recombination and coverage profiles

A genome-wide recombination profile is generated at the end of the process in which the parent and the recombinant genomes are plotted in parallel, and the alleles are represented in different colors according to their allele frequency status. The invariable genotypes (same ratio as the parent) are represented by gray markers (vertical lines), LOHs: ratio = 0, ratio = 1 are represented by red and blue markers, respectively and the heterozygous recombination events s are represented by black markers (e.g. 0.33–0.66 the marker at this location is black). To help identify chromosomal copy changes, the normalized depth of coverage of each chromosome is represented in parallel to the allele frequency where orange circles denote potential chromosomal loss and green dots denote potential chromosomal gain.

## Results

We developed a computational approach to detect regions of recombination characterized by variation of the allelic ratio. It is based on the likelihood distribution of the alleles (Additional file 1: Table S3) between a parental cell and its progeny or between two evolved strains regardless of the ploidy of the cells. First, all the Paired-end Illumina Fastq reads data were processed by Galocal.sh. The NGS reads from the 4-spores tetrad, and the diploid RTGs strains [11] were mapped to both S288C and SK1 genome reference separately, whereas the triploid and tetraploid clinical strains [13] were mapped, to the S288C genome. The alignment outputs (BAM files) are involved to call variants and calculate the per base depth of coverage. Next, the UGDR and NDoC pipelines are implemented to inspect the ratio variation distribution, analyze the normalized depth of coverage for potential chromosomal insertion/deletion and plot the recombination profile.

Interestingly, the study of ratio distribution showed that the ratios are not exact values of 0.33, 0.67 and 1.0 for triploid strains and 0.25, 0.5, 0.75 and 1.0 for tetraploid strains as they likely experience a slight experimental deviation ($\theta$ Additional file 1: Fig. S2). For example, the ratio of the heterozygous alleles in the diploid strains are centered around the value of 0.5, suggesting that the ratio of heterozygous alleles in diploids should fold in [$0.5-\theta$, 0.5 + $\theta$]. A similar observation has been made for the heterozygous allele ratio values in the triploid and tetraploid strains (Additional file 1: Fig. S2). Moreover, we observed a tight overlapping between the ratio groups of $0.25\pm \theta$ and $0.33\pm \theta$ and the ratio groups of $0.67\pm \theta$ and $0.75\pm \theta$, suggesting that groups of alleles ratios might overlap and errors arise in ratio variation analysis. Therefore, for a better classification of the ratios and to bypass potential ratio variation analysis confounders, we defined the standard deviation ($\theta$) as half the distance between the two adjacent groups (between 0.25 and 0.33 or 0.67 and 0.75), which is equal to $\theta$ = 0.04 (*p* value < 0.05).

To examine UGDR’s ability to detect recombination regions, we analyzed three sets of yeast genome sequences: four spore-tetrad (RTG10M-1A, 1B, 1C and 1D), two diploid hybrids RTG cells (RTG17M a mother and RTG17D a daughter) and four clinical euploid strains (triploids: YJM1138 and YJM1140, tetraploids: YJM958 and YJM959—Additional file 1: Table S1).

**Fig. 2:**
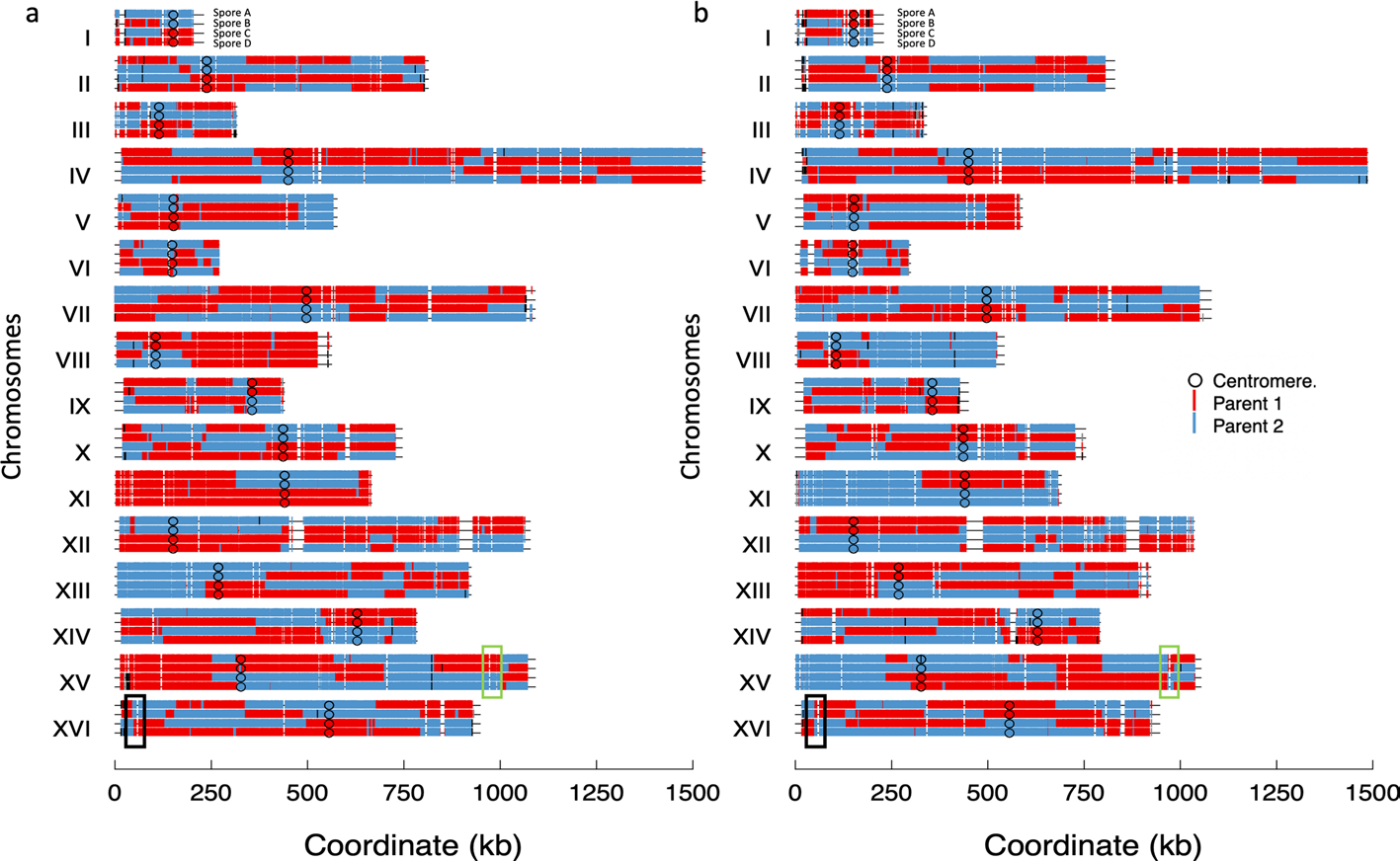
Segregation profile of four wild-type tetrads. The name of each tetrad is indicated on chromosome I. The x-axis represents the chromosome length and the y-axis represents the chromosome name. **a** The genotype of the tetrads when the S288C strain serves as reference, red markers are S288C (parent 1 S288C as a reference), blue markers are SK1 or parent 2 **b** The genotype of the tetrads when the SK1 strain serves as reference, red markers are SK1 (parent 1—SK1 as a reference), blue markers are S288C. Black and green boxes represent crossovers associated with (black boxes) or without (green box) an adjacent gene conversion segregate identified previously by [11]

The reads of the hybrid S288C/SK1 tetrads, were mapped to S288C (Fig. 2a) and SK1 (Fig. 2b) haploid references separately (reference genome of only one of the parents). Unsurprisingly, the recombination profile of the tetrads mapped to S288C mirrored the recombination profile of the tetrads mapped to SK1. Indeed, the allele (SNP) genotype is established when the genotype call matches the parental genome mapped against. For example, in Fig. 2a the red markers are the homozygous markers for S288C alleles (Parent 1) and the blue markers are the parent 2 (in this case it is SK1). On the other hand, in Fig. 2b the red markers are the homozygous markers for SK1 alleles (Parent 1) and the blue markers are the parent 2 (in this case it is S288C). This indicates that in case of hybrid the reference sequence of only one parent can be used to detect recombination of the progeny. Next, following the ratio variation analysis, the segregation profile of the four-spore tetrads exhibited interstitial and terminal LOH regions of variable length (Fig. 2). The segregation profile of most markers exhibited the expected Mendelian pattern: heterozygous alleles position exhibited 2:2 segregation pattern and the homozygous alleles position exhibited a 4:0 segregation pattern (beginning of chromosome XIII). Additionally, the regions identified previously as masked cross over with (black box) and without (green box) adjacent gene conversion have been identified.

**Fig. 3:**
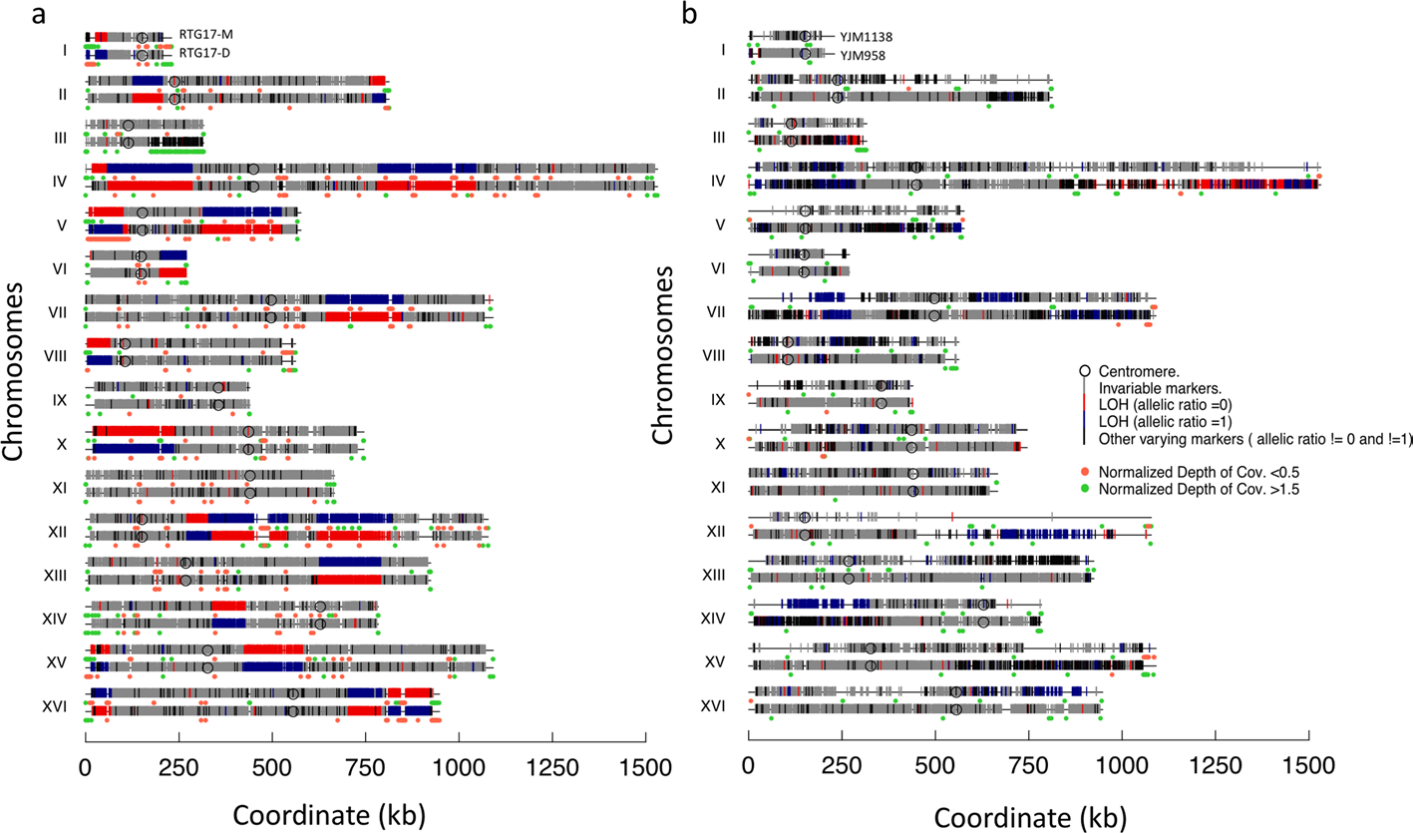
Genotype profile of diploids, triploid and tetraploid cells. **a** Genotype profile of the RTG17M and RTG17D cells mapped to S288C. **b** Genotype profile of polyploid strains: YJM1138 (3n) andYJM958 (4n). The genotype of the two RTGs and the genotype of 3n and 4n strains are shown on the top of each other (names are indicated on chromosome I). The x-axis represents the chromosome length and the y-axis represents the chromosome name. The orange and green dots represent the normalized depth of coverage (NDoC). Orange dots represent whether deletion occurs (NDoC < 0.5) and green dots represent whether insertion occurs (NdoC > 1.5). The genotypes: invariable (same ratio as the parent), LOH (ratio = 0), LOH (ratio = 1) and heterozygous recombination events are represented by the gray, red, blue and black markers (vertical lines), respectively

The reads of the RTG17M and RTG17D were also mapped to S288C and SK1 separately. Like the tetrads, the recombination profile of the diploid RTGs mapped to S288C mirrored the recombination profile of the RTGs mapped to SK1 (Fig. 3a, and Additional file 1: Fig. S3), indicating that the likelihood distribution of the allele frequency adopted by UGDR could be detected without a merged reference genome. Besides, the genotype of the diploids RTG17M and RTG17D (Fig. 3a) showed the expected opposite homozygote genotype supporting the occurrence of LOH regions in a successful return to growth process [11]. To note, the genotype profile of the RTG17D exhibits an increase of depth of coverage at the end of chromosomes III and a decrease of depth of coverage at the beginning of chromosomes V, indicating the presence of a duplication and deletion at these locations. These terminal chromosomal duplications/deletions were reported as a rearrangement resulting from recombination between dispersed Ty elements, that may result from ectopic Break Induced Replication (BIR) [20, 21]. In Laureau et al. analysis, the recombination profile of the RTG17D at these locations showed a heterozygous allele profile, but the depth of coverage analysis indicated the presence of chromosomal duplications/deletions. Interestingly, this event clearly detected by UGDR and visualized by the presence of black markers at the end of chromosome III indicating allele frequency changes along with an increase of the Normalized DoC (Fig. 3a, green dots). The duplication present at the end of chromosome III increases its ploidy from 2n to 3n, and as a result, the ratio of the embedded SNPs varied from 0.5 to 0.33 and/or 0.66 visible by the black bars at the end of chromosome III. However, the deletion observed on chromosome V (ploidy 2n to 1) is unambiguously distinguishable only by the decrease of the normalized DoC, since the SNPs ratio is either 1 (blue) or 0 (red). These results demonstrate the ability of UGDR, by combining the allele frequency variation and depth of coverage, to detect both LOH and structural variations missed by previous methods.

Lastly, the reads of the phylogenetically close strains (YJM1138, YJM1140, YJM959 and YJM958) were mapped to the S288C strain (close ancestry) and variant calling analysis retained > 68K heterozygous polymorphic positions along the 16 chromosomes for the recombination analysis. Since the parent of those cells is unknown, we selected for the triploids, the YJM1140 strain as a potential parent of the YJM1138 strain, and for the tetraploids, the strain YJM959 as a potential potential parent of the YJM958 strain. Although these strains were not the product of any recombination events, the analysis of ratio variation revealed the presence of several regions of recombination in the stains YJM1138, YJM958 relative to their selected parents. Regions where the recombination event led to large LOHs (Fig. 3b red markers: 0.33–0 and blue markers 0.66 to 1) and regions where the recombination events occur between two chromosomal copies leading to alleles ratio variation (e.g. 0.3–0.66 or 0.66–0.33) without inducing LOH (Fig. 3b Black markers—Additional file 1: Fig. S1).

## Discussion

In this study, we developed a python-based method called UGDR to detect recombination in different euploid strains and identify new out-performing recombinant yeast. To overcome the limitations of reference specificity and limited ploidy, UGDR is based uniquely on the analysis of allele ratio variation and supported by normalized depth of coverage. The allele ratio is calculated from the data obtained from the variant calling analysis, and the likelihood distribution of the allele frequency is used to detect recombination between two strains (parent and tetrads, mother and daughter or two phylogenetically close strains). The performance of UGDR was evaluated using four meiotic tetrads, two meiotically induced diploids, two triploids and two tetraploids obtained from the literature.

All previous computational approaches succeeded in detecting recombination in tetrads and diploids. Indeed, ReCombine [9] was designed to detect recombination in tetrads and requires a merged reference genome containing both parental genomes with some hard coded parameters making it hard to apply for tetrads of unknown reference genome. Recently, RecombineX [10], a general automatic framework for polymorphic markers identification, improved the process of gametes genotyping in yeasts and extended the process to green alga. Clearly, UGDR is more suitable for tetrads with unavailable merged reference parent or a parent different than S288C; nevertheless, RecombineX would be more appropriate to analyze genomes from other organisms. The recombination events reported by Laureau et al. [11] were detected based on  a merged reference. This strategy succeeded in screening the hybrid S288C/SK1 diploid RTGs but missed the direct detection of chromosomal rearrangement. Moreover, this method can’t be applied for other hybrids and ploidies. Meanwhile, UGDR was successful in accurately detecting recombination using separate parent and identifying both the presence of an ectopic recombination event and the change in the depth of coverage that occurred on the chromosomes III and V of the RTG17D. Additionally, UGDR was also able to screen different hybrid RTGs from two different industrial yeasts, different than S288C and SK1 (unpublished data). By extending its usage to polyploid strains, unlike previous methods, UGDR was successful in analyzing and accurately detecting recombination events in triploid and tetraploid yeasts downloaded from the literature. Several industrial triploids RTGs that underwent RTGs to induce recombination were analyzed by UGDR and the tool successfully selected different recombinants (with and without ploidy changes) that were later tested for their fermentation performance (unpublished data).

In conclusion, While UGDR is limited to detecting recombination regions in only yeast genomes with ploidy ≤ 4n, It provides information on the potential recombinations and/or chromosomal rearrangement that occurred between progenies and their parent, a mother and daughter and, unprecedentedly, between two polyploid cells. Such information helped in the identification of original recombinants that might be useful for their industrial application. Moreover, UGDR would be helpful for yeast’s new biotechnological research and applications with great benefits to humans [22, 23]. Further developments are aimed at including the analysis of genomes different than yeast and higher ploidies (> 4) in the perspective of expanding its use to other industries and academic research.

## Supplementary Information

**Additional file 1.** UGDR-Supplementary.

## Data Availability

UGDR is freely distributed under GNU General Public License and available at: https://github.com/AnimaTardeb/Meiogenix-UGDR. UGDR relies on the VCF files obtained by FreeBayes during variant calling and is compatible with Python version 2.7 (or later versions). The plotting programs are written in R and are compatible with R Version 3.2.3 or higher.
