# Supplementary material for: UGDR: a generic pipeline to detect recombined regions in polyploid and complex hybrid yeast genomes
Source: BMC Bioinformatics. 2022 Dec 21;23:555. doi: 10.1186/s12859-022-05113-y (PMC9773435; doi:10.1186/s12859-022-05113-y)
Supplement: Supplementary file 1 — Additional file 1. UGDR-Supplementary. [file 12859_2022_5113_MOESM1_ESM.pdf]

Additional materials

UGDR: A generic pipeline to detect recombined regions in polyploid and complex hybrid yeast genomes.

Amina Bedrat [1,2,3*]

[1]Institut Curie, 26 Rue d'Ulm, 75005 Paris, France.

[2]Meiogenix, 27 rue du Chemin Vert, 75011 Paris.

[3]Medical College of Wisconsin. WI, USA.

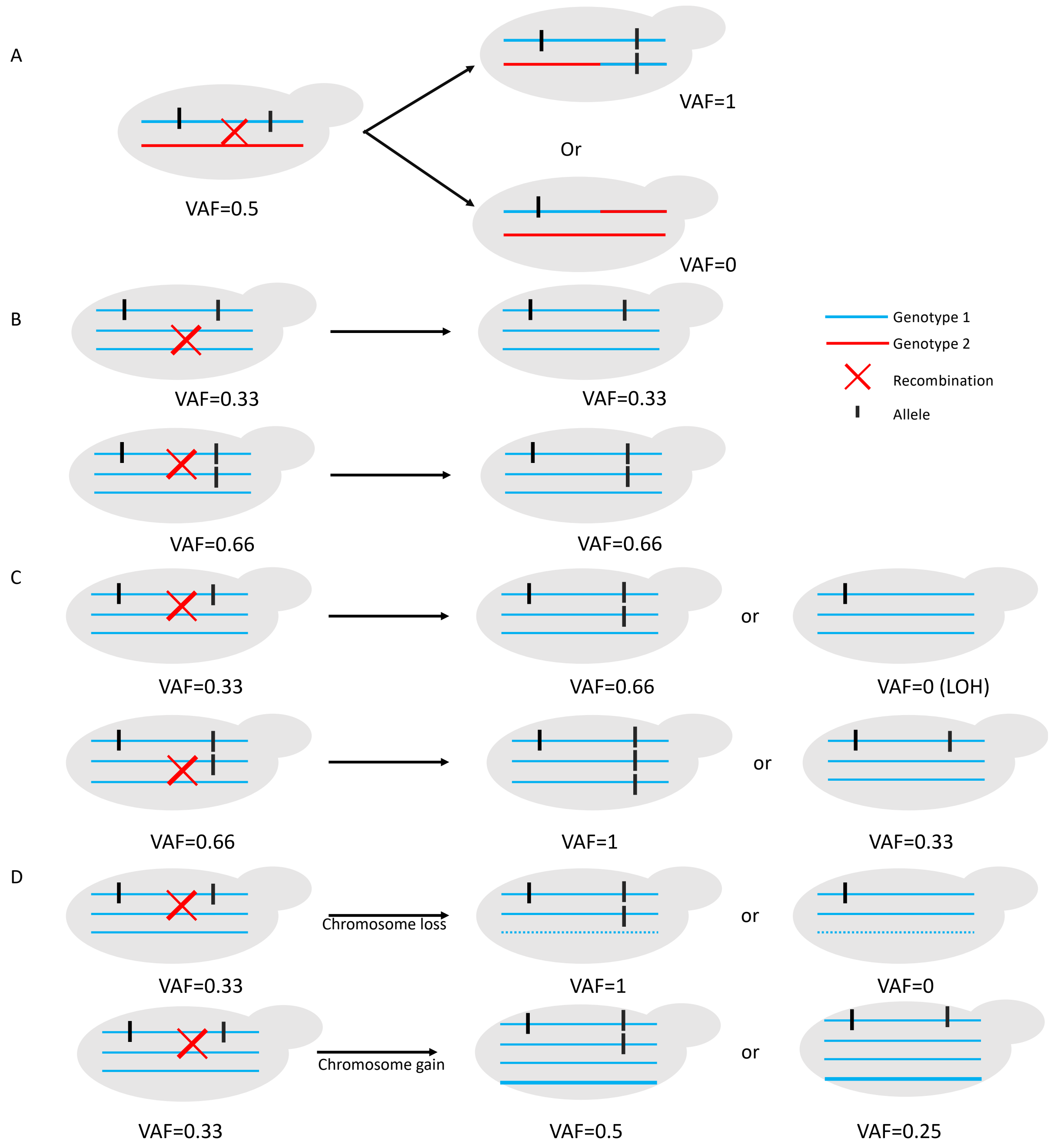

Figure S1: Summary of different allelic frequency variation during recombination upon yeast ploidy. (A) In diploids, heterozygous alleles recombine to form homozygous regions (LOH) toward one genotype or to another. (B) Masked recombination: in triploids the allelic frequency did not vary when recombination involves chromosome copies that did not carry the alleles (B-top: 1/3 →1/3) or both carry same allele (B-bottom: 2/3 → 2/3). (C) When recombination involves chromatids that one has an allele and the other one does not have the allele, the allelic frequency varies (1/3 → 0 (LOH) or 1/3 → 2/3 and 2/3 → 1/3 or 2/3 → 1(LOH)). (D) Recombination and chromosomal rearrangement followed by ploidy changes. LOH: Lost of heterozygosity. VAF: variant allele frequency.

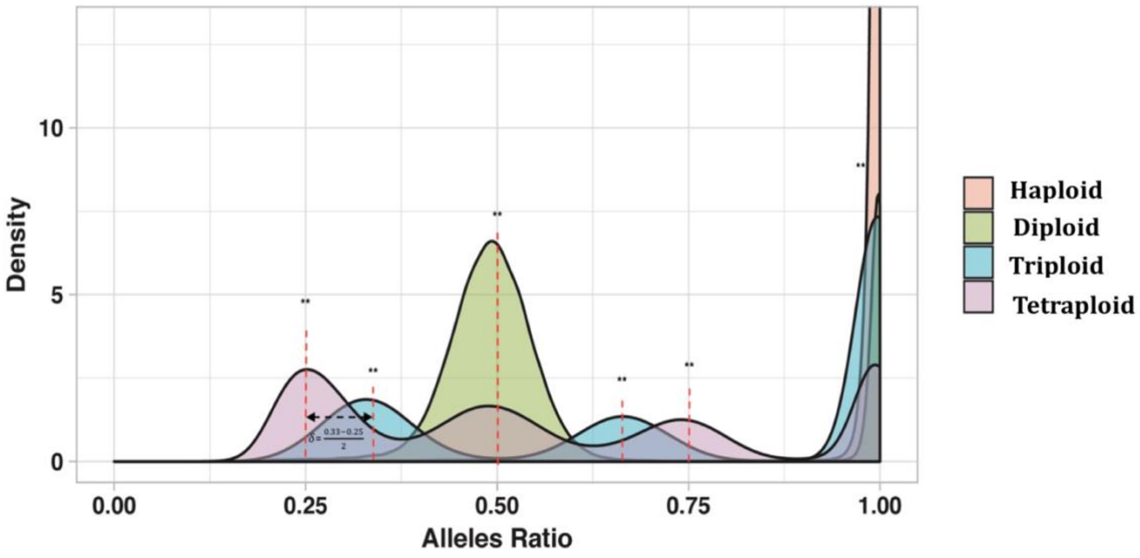

Figure S2: Distribution of the ratio among different ploidies.

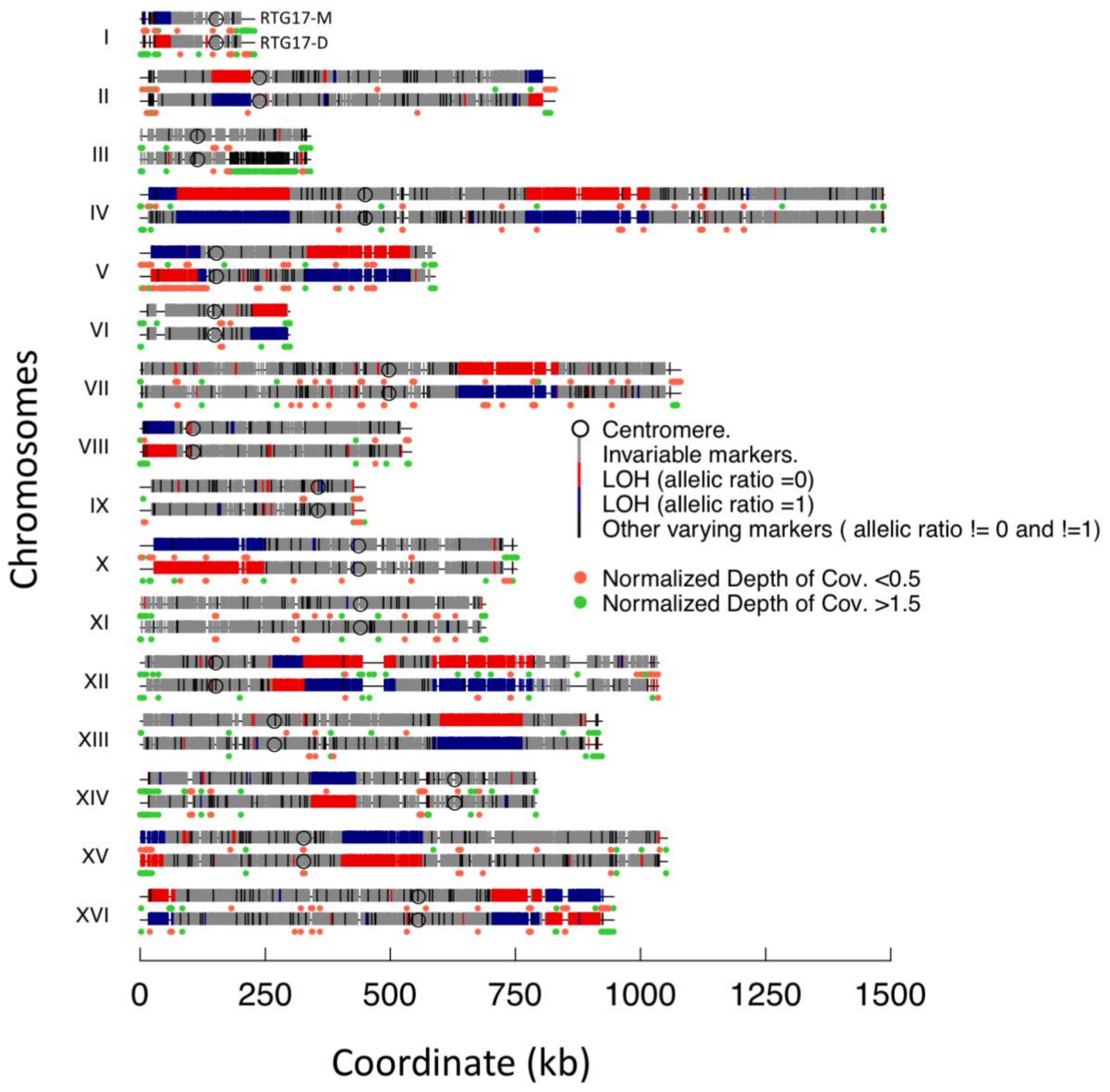

Figure S3: Genotype of the RTG17M and RTG17D when mapping to SK1. The genotype of the RTG17M and the RTG17D are shown on the top of each other (names are indicated in chromosome I), separated by dots representing the normalized depth of coverage (NDoC). Orange dots: if deletion occurs (NDoC <0.5) and green dots: if insertion occurs (NDoC >1.5). The genotypes: Invariable, LOH (ratio=0), LOH (ratio=1) and heterozygous recombination are represented by the gray, red, blue and black colored vertical lines.

NDoC: Normalized depth of coverage.

LOH: Lost of heterozygosity.

SRA Study	Names	Run	Sample Name	Unique ID		
SRP067222	SK1/S288C	SRR2984784	A102R49	AND1702 [*]	Hybrid S288c/SK1	(Laureau *et al.*, 2016)
	RTG17-M	SRR2984813	A143R13	AND2612 [**]		
	RTG17-D	SRR2984814	A143R14	AND2613 [**]		
	RTG10M-1A	SRR2984915	A102R13	AND1739-1A [***]		
	RTG10M-1B	SRR2984916	A102R14	AND1739-1B [***]		
	RTG10M-1C	SRR2984917	A102R15	AND1739-1C [***]		
	RTG10M-1D	SRR2984918	A102R16	AND1739-1D [***]		
SRP072079	2-134	SRR3265371	SRS1351586	YJM1138 [#]	*Saccharomyces cerevisiae*	(Zhu *et al.*, 2016)
	2-136	SRR3265373	SRS1351584	YJM1140 [#]		
	2-066	SRR3265444	SRS1351640	YJM958 ¶		
	2-067	SRR3265445	SRS1351665	YJM959 ¶		

Table S1: The different yeast used for recombination study. The 4 spores and the diploid mother and daughter RTGs are compared to the parental strain AND1702. The triploids are compared against each other. Similarly, for the tetraploids.

*: parental control strain (2n).
**: RTG_WT (2n).
***: Wild type spores.
# : Triploid strains (3n)
¶ : Tetraploid strains (4n)

Frequency	Haploid	Diploid	Triploid	Tetraploid
Heterozygous alleles	1	1/2	1/3	1/4
			2/3	1/2
				3/4
Homozygous alleles	-	1	1	1

Table S2: Distribution of the allelic ratio following the ploidy.

Allelic frequency (ratio)	Diploid	Triploid		Tetraploid			
**Parental ratio**	1/2	1/3	2/3	1/4	1/2	3/4	
**Invariant ratio**	1/2	1/3	2/3	1/4	1/2	3/4	**Recombined cell**
**Variant ratio**	0	2/3	1/3	1/2	1/4	1/2	
	1*	0*	1*	0*	3/4	1*	
					0*		

Table S3: potential allelic ratio variation. e.g.: in the parent/reference, the ratio of an allele x is 2/3, if in the recombined cell the ratio is still 2/3, x is categorized as an invariant allele and if the ratio varies to 1/3 or 1, x is then categorized as a variant allele group. * represent all the variation that are considered as LOH.

`
